# Supplementary material for: COZOID: contact zone identifier for visual analysis of protein-protein interactions
Source: BMC Bioinformatics. 2018 Apr 6;19:125. doi: 10.1186/s12859-018-2113-6 (PMC5889581; doi:10.1186/s12859-018-2113-6)
Supplement: Supplementary file 2 — Software build. Executable binary file of the software tool. (ZIP 118,784 kb) [file 12859_2018_2113_MOESM2_ESM.zip › cozoid/java/docs/beansbinding-1.2.1-doc.zip!/org/jdesktop/el/impl/parser/SimpleNode.html]

SimpleNode (Beans Binding)


---


|  |  |  |
| --- | --- | --- |
| **Overview** \| **Package** \| **Class** \| **Use** \| **Tree** \| **Deprecated** \| **Index** \| **Help** | |  |
| **PREV CLASS**   **NEXT CLASS** | **FRAMES**    **NO FRAMES**     **All Classes** |  |
| SUMMARY: NESTED \| FIELD \| CONSTR \| METHOD | DETAIL: FIELD \| CONSTR \| METHOD |  |


---


## org.jdesktop.el.impl.parser Class SimpleNode

```
java.lang.Object
  org.jdesktop.el.impl.lang.ELSupport
      org.jdesktop.el.impl.parser.SimpleNode
```

**All Implemented Interfaces:**: Node

**Direct Known Subclasses:**: ArithmeticNode, AstBracketSuffix, AstChoice, AstCompositeExpression, AstDeferredExpression, AstDotSuffix, AstDynamicExpression, AstEmpty, AstFloatingPoint, AstFunction, AstIdentifier, AstInteger, AstLiteralExpression, AstNegative, AstNot, AstNull, AstString, AstValue, BooleanNode

---

``` public abstract class SimpleNode extends ELSupport implements Node ```

---

| **Field Summary** | |
| --- | --- |
| `protected  Node[]` | `children` |
| `protected  int` | `id` |
| `protected  java.lang.String` | `image` |
| `protected  Node` | `parent` |


**Constructor Summary**

`SimpleNode(int i)`


| **Method Summary** | |
| --- | --- |
| `void` | `accept(NodeVisitor visitor)` |
| `void` | `dump(java.lang.String prefix)` |
| `java.lang.String` | `getImage()` |
| `MethodInfo` | `getMethodInfo(EvaluationContext ctx, java.lang.Class[] paramTypes)` |
| `java.lang.Class` | `getType(EvaluationContext ctx)` |
| `java.lang.Object` | `getValue(EvaluationContext ctx)` |
| `java.lang.Object` | `invoke(EvaluationContext ctx, java.lang.Class[] paramTypes, java.lang.Object[] paramValues)` |
| `boolean` | `isReadOnly(EvaluationContext ctx)` |
| `void` | `jjtAddChild(Node n, int i)`             This method tells the node to add its argument to the node's list of children. |
| `void` | `jjtClose()`             This method is called after all the child nodes have been added. |
| `Node` | `jjtGetChild(int i)`             This method returns a child node. |
| `int` | `jjtGetNumChildren()`             Return the number of children the node has. |
| `Node` | `jjtGetParent()` |
| `void` | `jjtOpen()`             This method is called after the node has been made the current node. |
| `void` | `jjtSetParent(Node n)`             This pair of methods are used to inform the node of its parent. |
| `void` | `setImage(java.lang.String image)` |
| `void` | `setValue(EvaluationContext ctx, java.lang.Object value)` |
| `java.lang.String` | `toString()` |
| `java.lang.String` | `toString(java.lang.String prefix)` |

**Methods inherited from class org.jdesktop.el.impl.lang.ELSupport**

`coerceToBoolean, coerceToCharacter, coerceToEnum, coerceToNumber, coerceToNumber, coerceToNumber, coerceToNumber, coerceToString, coerceToType, compare, containsNulls, equals, isBigDecimalOp, isBigIntegerOp, isDoubleOp, isDoubleStringOp, isLongOp, isStringFloat, throwUnhandled, toFloat, toNumber`

**Methods inherited from class java.lang.Object**

`clone, equals, finalize, getClass, hashCode, notify, notifyAll, wait, wait, wait`

**Field Detail**

### parent

```
protected Node parent
```

---


### children

```
protected Node[] children
```

---


### id

```
protected int id
```

---


### image

```
protected java.lang.String image
```


**Constructor Detail**

### SimpleNode

```
public SimpleNode(int i)
```


**Method Detail**

### jjtOpen

```
public void jjtOpen()
```

:   **Description copied from interface: `Node`**
:   This method is called after the node has been made the current
    node. It indicates that child nodes can now be added to it.

    :   **Specified by:**: `jjtOpen` in interface `Node`

---


### jjtClose

```
public void jjtClose()
```

:   **Description copied from interface: `Node`**
:   This method is called after all the child nodes have been
    added.

    :   **Specified by:**: `jjtClose` in interface `Node`

---


### jjtSetParent

```
public void jjtSetParent(Node n)
```

:   **Description copied from interface: `Node`**
:   This pair of methods are used to inform the node of its
    parent.

    :   **Specified by:**: `jjtSetParent` in interface `Node`

---


### jjtGetParent

```
public Node jjtGetParent()
```

:   **Specified by:**: `jjtGetParent` in interface `Node`

---


### jjtAddChild

```
public void jjtAddChild(Node n,
                        int i)
```

:   **Description copied from interface: `Node`**
:   This method tells the node to add its argument to the node's
    list of children.

    :   **Specified by:**: `jjtAddChild` in interface `Node`

---


### jjtGetChild

```
public Node jjtGetChild(int i)
```

:   **Description copied from interface: `Node`**
:   This method returns a child node. The children are numbered
    from zero, left to right.

    :   **Specified by:**: `jjtGetChild` in interface `Node`

---


### jjtGetNumChildren

```
public int jjtGetNumChildren()
```

:   **Description copied from interface: `Node`**
:   Return the number of children the node has.

    :   **Specified by:**: `jjtGetNumChildren` in interface `Node`

---


### toString

```
public java.lang.String toString()
```

:   **Overrides:**: `toString` in class `java.lang.Object`

---


### toString

```
public java.lang.String toString(java.lang.String prefix)
```

---


### dump

```
public void dump(java.lang.String prefix)
```

---


### getImage

```
public java.lang.String getImage()
```

:   **Specified by:**: `getImage` in interface `Node`

---


### setImage

```
public void setImage(java.lang.String image)
```

---


### getType

```
public java.lang.Class getType(EvaluationContext ctx)
                        throws ELException
```

:   **Specified by:**: `getType` in interface `Node`
:   **Throws:**: `ELException`

---


### getValue

```
public java.lang.Object getValue(EvaluationContext ctx)
                          throws ELException
```

:   **Specified by:**: `getValue` in interface `Node`
:   **Throws:**: `ELException`

---


### isReadOnly

```
public boolean isReadOnly(EvaluationContext ctx)
                   throws ELException
```

:   **Specified by:**: `isReadOnly` in interface `Node`
:   **Throws:**: `ELException`

---


### setValue

```
public void setValue(EvaluationContext ctx,
                     java.lang.Object value)
              throws ELException
```

:   **Specified by:**: `setValue` in interface `Node`
:   **Throws:**: `ELException`

---


### accept

```
public void accept(NodeVisitor visitor)
            throws ELException
```

:   **Specified by:**: `accept` in interface `Node`
:   **Throws:**: `ELException`

---


### invoke

```
public java.lang.Object invoke(EvaluationContext ctx,
                               java.lang.Class[] paramTypes,
                               java.lang.Object[] paramValues)
                        throws ELException
```

:   **Specified by:**: `invoke` in interface `Node`
:   **Throws:**: `ELException`

---


### getMethodInfo

```
public MethodInfo getMethodInfo(EvaluationContext ctx,
                                java.lang.Class[] paramTypes)
                         throws ELException
```

:   **Specified by:**: `getMethodInfo` in interface `Node`
:   **Throws:**: `ELException`


---


|  |  |  |
| --- | --- | --- |
| **Overview** \| **Package** \| **Class** \| **Use** \| **Tree** \| **Deprecated** \| **Index** \| **Help** | |  |
| **PREV CLASS**   **NEXT CLASS** | **FRAMES**    **NO FRAMES**     **All Classes** |  |
| SUMMARY: NESTED \| FIELD \| CONSTR \| METHOD | DETAIL: FIELD \| CONSTR \| METHOD |  |


---
